# The current status of risk-stratified breast screening

**DOI:** 10.1038/s41416-021-01550-3

**Published:** 2021-10-26

**Authors:** Ash Kieran Clift, David Dodwell, Simon Lord, Stavros Petrou, Sir Michael Brady, Gary S. Collins, Julia Hippisley-Cox

**Affiliations:** 1 Cancer Research UK Oxford Centre, Department of Oncology, University of Oxford, Oxford, UK; 2 Nuffield Department of Primary Care Health Sciences, University of Oxford, Oxford, UK; 3 Nuffield Department of Population Health, University of Oxford, Oxford, UK; 4 Department of Oncology, University of Oxford, Oxford, UK; 5 Centre for Statistics in Medicine, Nuffield Department of Orthopaedics, Rheumatology & Musculoskeletal Sciences, University of Oxford, Oxford, UK; 6 NIHR Oxford Biomedical Research Centre, John Radcliffe Hospital, Oxford, UK

**Keywords:** Breast cancer, Cancer epidemiology

## Abstract

Apart from high-risk scenarios such as the presence of highly penetrant genetic mutations, breast screening typically comprises mammography or tomosynthesis strategies defined by age. However, age-based screening ignores the range of breast cancer risks that individual women may possess and is antithetical to the ambitions of personalised early detection. Whilst screening mammography reduces breast cancer mortality, this is at the risk of potentially significant harms including overdiagnosis with overtreatment, and psychological morbidity associated with false positives. In risk-stratified screening, individualised risk assessment may inform screening intensity/interval, starting age, imaging modality used, or even decisions not to screen. However, clear evidence for its benefits and harms needs to be established. In this scoping review, the authors summarise the established and emerging evidence regarding several critical dependencies for successful risk-stratified breast screening: risk prediction model performance, epidemiological studies, retrospective clinical evaluations, health economic evaluations and qualitative research on feasibility and acceptability. Family history, breast density or reproductive factors are not on their own suitable for precisely estimating risk and risk prediction models increasingly incorporate combinations of demographic, clinical, genetic and imaging-related parameters. Clinical evaluations of risk-stratified screening are currently limited. Epidemiological evidence is sparse, and randomised trials only began in recent years.

## Introduction

Breast screening is widely implemented in many healthcare systems to reduce breast cancer mortality through the expedited diagnosis of smaller, asymptomatic breast cancers. For the majority of women, this uses mammography starting in middle age, although different regions differ in their screening recommendations and practices (Table 1). In rarer, very high-risk situations such as a known, high-penetrance genetic predisposition, earlier screening with magnetic resonance or ultrasound imaging is advocated [1, 2].

**Table 1:** Summary of national screening programme strategies or national body recommendations for screening women who are not at elevated risk of breast cancer (e.g. those without a known familial risk/genetic predisposition, or history of chest wall radiotherapy).

Country	Age group	Screening strategy
United Kingdom (NHS Breast Screening Programme) [161]	Women aged 50–70 Women aged 71 years and older	Invitation to mammography screening every 3 years Not invited—may self-refer
United States of America (United States Preventive Service Task Force) [162]	Women aged 40–49 years Women aged 50–74 years Women aged 75 years and older	Individual decision-making recommended Biennial mammography No recommendation: evidence insufficient to assess harms and benefits in this age group
Canada (Canadian Task Force on Preventive Health Care) [163]	Women aged 40–49 years Women aged 50–69 years Women aged 70–74 years	Not recommended; shared decision-making if desired Mammography every 2–3 years Mammography every 2–3 years
Netherlands (National Breast Cancer Screening Programme) [164]	Women aged 50–75 years	Invitation to mammography every 2 years
Australia (BreastScreen Australia) [165]	Women aged 40–49 years Women aged 50–74 years Women aged 74 years and older	Not invited, but may ‘opt-in’ Invitation to mammography every 2 years Not invited but may ‘opt-in’
China (National Health Commission of the People’s Republic of China) [166]	Women aged 20–39 years Women aged 40–69 years Women aged 70 years and older	Monthly breast self-examination, clinical breast examination 1–3 yearly Mammography every 1–2 years with ultrasound for women with dense breasts; monthly breast self-examination and annual clinical breast examination Monthly breast self-examination, annual clinical breast examination

Whilst meta-analysis of randomised clinical trial data clearly demonstrates a reduction in the relative risk of breast cancer mortality due to screening, reduced breast cancer deaths may come at the expense of overdiagnosis (the identification and unnecessary treatment of clinically insignificant tumours), as well as the consequences of false-positive or false-negative results [3–8]. There is wide variability in the results seen in observational studies in relation to overdiagnosis estimations, depending on analytical or modelling approaches [9–12]. The summation of evidence assessed by the UK Independent Panel [3] was that breast screening does reduce mortality. It concluded that for every 10,000 women in the United Kingdom aged 50 years invited to screening for the next 20 years, 43 breast cancer deaths would be avoided and 681 tumours (invasive or ductal carcinoma in situ [DCIS]) would be diagnosed, but 129 women would be overdiagnosed, i.e. three overdiagnosed cases per breast cancer death averted, although this calculated benefit–harm balance has been contested by some [13, 14].

Most countries use an age-based population-level breast screening strategy that reduces breast cancer mortality but does not account for the wide variation in individual women’s cancer risks [15–18]. Identification of women at the highest risk of breast cancer may enable targeted intensification of early detection or preventive measures, and reduce the public health burden of this common malignancy. Motivated by such factors, ‘risk-stratified’ breast screening has emerged as a concept in which decisions to offer screening or the determination of screening frequency and modality (e.g. from mammography/tomosynthesis to magnetic resonance imaging [MRI]) are guided by accurate estimation of an individual woman’s risk of breast cancer [15, 19–21]. Logically, risk-stratified screening would target intervention at those that stand to gain the most and reduce or stop screening in those that stand to gain little benefit, potentially also informed by the cost-effectiveness evidence.

However, the efficacy and feasibility of personalising screening strategies is uncertain and would require the meeting of several critical dependencies for implementation [21]. These include the need for rigorously developed and validated risk prediction models capable of stratifying women accurately, robust health economic evaluation, and clarity on the balance sheet of benefits and harms that would become the ‘new norm’ in clinical practice after prospective studies. Although some recent reviews have sought to summarise this field [21–23], they differ in the extent to which they cover each of these dependencies. In this narrative scoping review, the authors seek to provide a concise overview of the key topics within the risk-stratified screening literature and anticipate the potential effects of emerging evidence on clinical practice. First, the evidence for risk prediction modelling that may guide personalised screening is reviewed. Thereafter, the evidence from observational analyses of epidemiological or registry data is appraised prior to discussion of how ongoing prospective cohorts and trials have been designed in this area. Furthermore, health economic evidence and output from qualitative studies is synthesised. Throughout, there is an emphasis on evidence quality, its limitations and discussion of how unmet needs may be satisfied.

## Methods

A scoping literature review was undertaken using Medline (PubMed) with the following search strategy: (“breast screening” OR “mammography”) AND (“risk#adapted” OR “risk#stratified” OR “personalised” OR “personalized” OR “tailored” OR “risk#based”). Papers published in any language prior to 1 November 2020 were considered for inclusion in this review. We reviewed the reference lists of systematic reviews to identify key publications if not identified by the search strategy. We also searched clinicaltrials.gov on 1 November 2020 to identify ongoing interventional studies in this area (for search terms: “breast cancer”, “screening” and “risk”).

Reports retrieved were screened for inclusion based on title and abstract and, if relevant, were classified into five groups: papers reporting risk prediction models, epidemiological analyses of risk-stratified screening or retrospective evaluations, prospective studies and trials of risk-stratified screening, health economic evaluations and qualitative research on feasibility or acceptability. Findings were synthesised narratively, informed by the narrative synthesis guidelines developed by the Cochrane Collaboration [24].

### Risk prediction models to guide personalised screening

Several risk prediction models for breast cancer incidence have been reported, which tend to incorporate ‘classical’ risk factors identified from epidemiological evidence, e.g. clinical, demographic or pharmacological exposures, but may also assimilate factors such as family history, genetic risk markers or polygenic risk scores and imaging-related parameters. Well-recognised ‘risk factors’ include breast density, first-degree family history of breast cancer, increased body mass index (BMI), nulliparity or young age at first birth, and such factors may be attributable for over 52% of risk [25]. The role of obesity relative to menopause, such as the apparent ‘protective effect’ of obesity on oestrogen receptor-positive cancers prior to menopause [26, 27], may be relevant but is not always incorporated into risk models [28].

Some models have also sought to incorporate markers of genetic risk, such as the inclusion of *BRCA* genotype in the Tyrer–Cuzick (also known as the ‘IBIS’) model [29], as well as the incorporation of a polygenic risk estimation incorporated into a later update thereof [30]. Some comprise predominantly genetic information, such as BOADICEA [31–33] and BRCAPRO [32, 34]. Genetic predisposition, through either highly penetrant mutations such as *BRCA1/2* [35, 36] or subtler single-nucleotide polymorphisms (SNPs) may indeed affect breast cancer risk; however, only 25–50% of the familial risk can be explained by known genetic variants [35, 37–40], and only 16% of the risk of nonfamilial breast cancer is accounted for by SNPs [36]. Furthermore, breast density and textural features [41] may be relevant to breast cancer risk and have been explored as covariates in risk prediction models rooted in mammographic image analysis or as additions to covariate panels in updated versions of statistical models. Generally, assessment of breast density could be determined by visual assessment scales [42], or automated/algorithmic approaches [43–45], but the most common appears to be the four-category ‘Breast Imaging Reporting and Data System’ classification [46] (BI-RADS) [30, 47, 48]. Modelling approaches utilised include mathematical modelling [29], statistical regression [49, 50] or ‘machine learning’ techniques [51].

Whilst the implementation of risk-stratified screening to entire populations depends on the prospective outcome and economic evaluations, the requirement for accurate and robust multivariable risk prediction models is the *sine qua non* of any such approach [52]. All risk prediction models intended to be used to inform clinical decision-making should be transparently reported, and robustly evaluated in terms of various performance metrics [53]. Strong internal validation is recommended, and appropriate external validation using data sources that are independent of those used to generate the model may also be useful [54, 55]. Important considerations include the discrimination of models, i.e. how well they distinguish between women who do develop breast cancer versus those who do not [56]; calibration, i.e. the degree of agreement between the predicted risks and the observed risks [57]; and assessment of ‘net benefit’ using decision-curve analysis [58, 59]. In terms of discrimination, the ‘area under the curve’ (AUC) or identical ‘*c*-statistic’ may be used for binary outcomes, or the ‘*c*-index’ for survival data [60] both range between 0 and 1, with values of 1 corresponding to perfect prediction and 0.5 reflecting discrimination no better than a coin toss. Other metrics to consider include the proportion of variance explained by the model, such as the R^2^ [2, 61]. It is increasingly clear that average performance metrics or ‘overall’ assessments of model performance in populations are insufficient to truly assess clinical utility on deployment, as there may be differences in model performance between regions, ethnic groups or even age groups [62, 63].

Table 2 describes the development and validation results regarding key published risk prediction models. Our search strategy sought to identify original reports and secondary validation studies without restricting the latter to those performed by model developers. Interestingly, a recent systematic review by Louro et al. [64], which appraised the evidence for the Breast Cancer Risk Assessment Tool (BCRAT), Breast Cancer Surveillance Consortium (BCSC), Rosner and Colditz, IBIS and other models using the ISPOR-AMCP-NPC [65] questionnaire rather than PROBAST [66] found that it was challenging to recommend any model for the purposes of risk-stratified screening. Importantly, some risk prediction models were missed by their search strategy [50, 67], and models focussing on genetic determinants of risk were intentionally excluded [64]. The emergent pattern is that incorporation of multiple data forms, such as adding breast density or other mammographic features, or genetic information yields incremental gains in model performance [68], although these tend to be relatively small [30, 69]. Specifically, regarding incorporating breast density on discrimination, the increase in the AUC of published models ranges from 0.03 to 0.14 [69]. The effects on calibration are less clear and the effects on net benefit are not reported.

**Table 2:** Details regarding study data, modelling strategy and performance metrics of notable published risk prediction models for breast cancer or their ‘updates’ identified during the scoping review.

Study first author and year	Study design and setting	Risk trajectory modelled	Study participants (*n*) and cases	Risk model covariates/data type combinations	Validation strategy	Discrimination metrics reported (95% confidence interval)	Calibration metrics reported (95% confidence interval)	Examination of performance heterogeneity
‘Tyrer–Cuzick’ or ‘IBIS’ model and updates or variations								
Tyrer et al. [29]	Construction of the model from first principles/informed by other published data	Diagnosis of breast cancer	N/A	Age, *BRCA* genotype, family history of breast cancer, (including relationship and ages), menarche, age at first birth, menopausal status, atypical hyperplasia, lobular carcinoma in situ, height, BMI	None	None	None	None
Brentnall et al. [47]	Cohort study (Kaiser Permanente Washington registry), US, women aged 40–73 years	Diagnosis of breast cancer within 19 years	132,139 (2699 breast cancer cases)	Tyrer–Cuzick model Tyrer–Cuzick model + breast density category	Evaluated pre-designed risk calculation mechanism in the whole cohort	Separation of cumulative risk curves for pre-determined risk groups Upper tenth, lower tenth and ‘middle’ 8 tenths compared with hazard ratios	O/E: 1.02 (0.98–1.06) O/E: 0.98 (0.94–1.02)	O/E of models by three age groups (<50, 50–59, 60+ years)
van Veen et al. [30]	Cohort study (PROCAS), England, women aged 46–73 years	Diagnosis of breast cancer within 10 years	9363 (466 cases, 196 incident)	Tyrer–Cuzick model T–C + mammographic density T–C + mammographic density + polygenic risk score (18 SNPs)	Evaluated pre-designed risk calculation mechanism in the whole cohort	AUC 0.58 (0.52–0.62) AUC 0.64 (0.60–0.68) AUC 0.67 (0.62–0.71)	O/E: 1.50 (1.33–1.70) O/E: 0.98 (0.69–1.28)	Discrimination and calibration in terms of tumour ER status, and by invasive/DCIS tumour type
‘Gail’ or ‘BCRAT’ model and updates or variations								
Gail et al. [167]	Case–control study, US, White women aged 50 years and over	Projected breast cancer probabilities within 10, 20 and 30 years of follow-up	5998 (2852 cases)	Age at menarche, age at first live birth, number of previous breast biopsies, number of first-degree relatives with breast cancer	None	None	None	None
Gail et al. [168]	Case–control study (‘CARE’), US, African-American women aged 35–64 years	Projected breast cancer probabilities within 10, 20 and 30 years of follow-up	3254 (1607 breast cancer cases)	As for BCRAT, re-fitted in data from African-American women	External validation Women’s Health Initiative cohort	*C*-index 0.555 (0.535–0.575)	O/E: 1.08 (0.97–1.20)	O/E ratio by age groups, age at menarche, number of biopsies, and number of first-degree relatives *c*-index by age groups
Tice et al. [169]	Cohort study, US, women aged 35 years and over	Diagnosis of invasive breast cancer (no explicit horizon, median follow-up 5.1 years)	81,777 (955 breast cancer cases)	BCRAT BCRAT + BI-RADS breast density category	Apparent performance in study dataset	*C*-index 0.67 (0.65–0.68) *C*-index 0.68 (0.66–0.70)	None	None
Zhang et al. [170]	Nested case–control study, US, women aged 34–70 years	Diagnosis of invasive breast cancer within 5 years	11,880 (4006 cases)	BCRAT risk score + polygenic risk score (67 SNPs) + mammographic density + estrone sulfate + testosterone + prolactin	Internal: cross-validation	All women: AUC 0.65 (0.64–0.66) Described 5-year risk per predicted risk percentile	None	Age group-specific changes in AUC with sequential addition of variables to model
BCSC model (Breast Cancer Surveillance Consortium)								
Tice et al. [171]	Cohort study, US, women undergoing mammography aged 35 years and over	Diagnosis of invasive breast cancer within 5 and 10 years	1,135,977 (17,908 breast cancer cases)	Age, race/ethnicity, family history of breast cancer, breast biopsy history, benign breast disease, BI-RADS breast density	Update of an earlier model developed using a sample of cohort [49], five-fold cross- validation	AUC 0.665	E/O ratio 1.04 (1.03–1.06)	E/O across 5-year age groups, ethnic groups, levels of predictor parameters
Vachon et al. [48]	Nested case–control study, US women undergoing mammography; two further case–control studies (US and Germany)	Diagnosis of invasive breast cancer within 5 years	1622 (set 1) 1529 (set 2) 879 (set 3) (total 1634 breast cancer cases)	BCSC + PRS (76 loci) + BI-RADS breast density	Evaluation of models formed using datasets 2 and 3 using dataset 1	AUC 0.69 (0.67–0.71)	Hosmer–Lemeshow test of calibration	None
Rosner–Colditz model and updates or variations								
Rosner and Colditz, 1996 [172]	Cohort study, US, Caucasian women aged 30–64 years	Diagnosis of breast cancer (no specific horizon)	89,132 (2249 breast cancer cases)	Age, age at menarche, age at menopause, age at first birth, age at subsequent births	None	None	None	None
Rosner et al. [173]	Cohort study, US, Caucasian women aged 30–64 years	Diagnosis of breast cancer (no specific horizon)	66,145 (1559 breast cancer cases)	Age, age at menarche, age at menopause, age at first birth, age at subsequent births, benign breast disease history, HRT use, first-degree family history of breast cancer, weight, BMI, alcohol intake, oestradiol levels	None (assessed fit of log-incidence model)	AUC 0.645	None	None
Zhang et al. [170]	Nested case–control study, US, women aged 34–70 years	Diagnosis of invasive breast cancer within 5 years	11,880 (4006 cases)	BCRAT risk score + polygenic risk score (67 SNPs) + mammographic density + estrone sulfate + testosterone + prolactin	Internal: cross-validation	All women: AUC 0.678 (0.666–0.690) Described 5-year risk per predicted risk percentile	None	Age group-specific changes in AUC with the sequential addition of variables to model
Other models								
Hippisley-Cox and Coupland 2015 [50]	Cohort study in primary care (England), women aged 25–84 years	Diagnosis of breast cancer within 10 years	3,318,258 (41,315 breast cancer cases)	Age, BMI, deprivation score, ethnicity, alcohol intake, family history of breast cancer, benign breast disease, oral contraceptive use, oestrogen-containing HRT use, manic depression/schizophrenia, previous blood cancer, previous lung cancer, previous ovarian cancer	Split-sample validation	*C*-index 0.761 (0.758–0.765) *D*-statistic 1.088 (1.058–1.119)	Calibration plots (by a tenth of predicted risk)	Performance by age groups
Pal Choudhury et al. (iCARE models) [77]	Cohort study in UK, women aged 35–74 years	Diagnosis of invasive breast cancer within 5 years	UK-based validation: 64,874 (863 breast cancer cases) US-based validation for iCARE-Lit: 47,279 (1008 breast cancer cases)	*iCARE-BPC3*: age at menarche, age at menopause, parity, age at first birth, height, alcohol intake, first-degree relative with breast cancer, ever/never smoker, BMI, HRT use (type), current HRT use, the interaction between BMI and HTY type *iCARE-Lit*: age at menarche, age at menopause, parity, age at first birth, OCP use, BMI, height, alcohol intake, benign breast disease history, first-degree family history of breast cancer, the interaction between BMI and HRT use	External validation of models	*UK:* AUC over 50 s: 0.602 (0.580–0.624) AUC under 50 s: 0.654 (0.621–0.687) AUC over 50 s: 0.622 (0.600–0.645)	Over 50 s: E/O: 1.00 (0.93–1.09) CS: 0.960 (0.680–1.239) CI: 0.001 (−0.004 to 0.005) Over 50 s: E/O 1.13 (1.04–1.22) CS: 0.811 (0.668–0.954) CI: 0.001 (−0.001 to 0.004)	Discrimination and calibration metrics by age groups (<50, 50+ years)
Ming et al. [51]	Single centre (oncogenetic unit), women aged 20–80 years, Switzerland	Lifetime risk of breast cancer (classified into <17, 17–29, 30+%)	112,587 (4911 breast cancer cases)	ML classification models: Markov chain Monte Carlo generalised linear mixed model, adaptive boosting and random forest Same inputs as BOADICEA	Internal: repeated cross-validation	ML models: AUC 0.843–0.889 BOADICEA: AUC 0.639	Not presented	None
Park et al. (KoBCRAT) [174]	Case–control study, Korea No age limits	5-year and lifetime risk of breast cancer	9248 (4601 breast cancer cases)	Same predictors as Gail model, re-developed in the Korean population	External validation in further Korean cohort studies	AUC 0.61 (0.49–0.72) in one cohort, AUC 0.89 (0.85–0.93 in the other)	E/O: 0.97 (0.67–1.40) in the first cohort, 0.96 (0.70–1.37) in second cohort	AUCs in women aged <50 and 50+ years
Abdolell et al. [175]	Nested case–control study from a population-based screening program in Canada, women aged 40–75 years	Risk of breast cancer diagnosis at mammography	7770 (1882 breast cancer cases)	Percentage mammographic density, breast volume, age, core biopsy history, first-degree family history, number of births, menopausal status, HRT use	Apparent performance in study dataset (no validation)	Imaging features model: AUC 0.597 Imaging features + biopsy history: AUC 0.660 Full model: AUC 0.665	Not presented	None
Wang et al. [176]	Systematic review to pool odds ratios from observational studies to produce a risk score. Evaluated in Chinese women	Risk of breast cancer within 5 years	62,875 (15 cases of breast cancer)	Age at menarche, age at first birth, benign breast disease history, family history of breast cancer, history of breastfeeding, number of terminations of pregnancy	Evaluated pre-designed risk calculation mechanism in the whole cohort	AUC 0.64 (0.50–0.78)	None	None
Ueda et al. [28]	Case–control study, Japanese women at a single institution	Risk of breast cancer within 10 years, 20 years, and until age 84 years	376 breast cancer patients (no information on the number of controls)	Age at menarche, age at first delivery, family history of breast cancer, BMI (if post-menopausal)	None	None	None	None
Eriksson et al. [93]	Nested case–control study, Swedish women aged 31–79 years undergoing screening	Risk of breast cancer within 2 years	2165 (433 cancer cases)	*(Different model weights for pre- and post-menopausal women):* BMI, HRT use, family history of breast cancer, breast density, absolute difference in density between breasts, absolute difference in microcalcifications between breasts, an interaction term for % breast density and masses	Cross-validation (number of folds not specified)	AUC 0.71 (0.69–0.73)	None	None
Barlow et al. [177]	Cohort study, US women aged 35–84 years that underwent a previous mammogram in the preceding 5 years	Risk of invasive breast cancer or DCIS following a screening mammogram within 1 year	1,007,600 (11,638 breast cancer cases)	*Pre-menopausal women:* age, breast density (BI-RADS category), family history of breast cancer, and a prior breast procedure *Postmenopausal women:* age, breast density, race/ethnicity, family history of breast cancer, prior breast procedure, BMI, natural menopause, HRT, prior false-positive mammogram	Split-sample validation	Pre-menopausal women: AUC 0.629 (0.603–0.656) Post-menopausal women: AUC 0.626 (0.615–0.637)	Hosmer–Lemeshow goodness of fit	None (separate models fitted for pre- and post-menopausal women)

In terms of discrimination metrics, the *c*-statistic or *c*-index is reported as the AUC and *c*-statistic are identical for binary outcomes, whereas *c*-index is specifically for survival/time-to-event data; we have named the metrics using the appropriate nomenclature dictated by the statistical approach used, regardless of the name used in the original publication. It is important to note that the AUC/*c*-indices for each model development paper are not directly comparable, as each study population varies by age, region and setting. The above information is intended as a narrative overview of extant models as they are reported in their respective study populations, and does not constitute a formal comparison of model performance.

*N/A* not applicable, *O/E ratio* observed to expected ratio, *E/O ratio* expected to observed ratio, *BI-RADS* Breast Imaging-Reporting and Data System Classification, *AUC* area under the receiver operating curve, *BMI* body mass index, *HRT* hormone replacement therapy, *ML* machine learning, *ER+* oestrogen receptor positive, *ER−* oestrogen receptor negative, *PR+* progesterone receptor positive, *PR−* progesterone receptor negative, *SNP* single-nucleotide polymorphisms, *CS* calibration slope, *CI* calibration intercept (i.e. calibration in the large).

It is important to note that simply comparing AUC/*c*-indices of different model development studies does not constitute a meaningful comparison as a breast cancer risk is strongly influenced by age, and the AUC/*c*-index may be influenced by the heterogeneity of study population, the prediction horizons used and the source of the population used may differ across studies. For example, the AUC/*c*-index of a model developed in a cohort with a very broad age range is not directly transferable to a separate cohort of women with a narrower age range, such as women eligible for screening currently.

The O/E ratio is widely used to assess calibration, and simply compares the overall number of observed cases versus the number predicted by a model for a given population, and as a standalone metric is insufficient, as over-prediction in sub-groups can be compensated by under-prediction in others and vice versa [57]. A more comprehensive analysis of alignment between predicted versus observed risks for individual study participants could include the use of a calibration plot displaying (mis)alignment across levels of risk [70]. Some papers report as one aspect of their analyses the hazard ratio of pre-selected highest risk groups (such as top tenth) to middle-risk groups (such as middle 80%) [47]. This alone may provide an incomplete assessment of model performance as it is comparing small groups at the extremes of a risk score distribution to the bulk of the study population, which would naturally be expected to diverge in their observed risk. These must therefore be interpreted in the context of the other sources of information regarding model performance when provided. A key external validation study of four key models in a cohort of 15,732 women from Australia, Canada and the US (519 cases of breast cancer) demonstrated *c*-statistics of 0.70 for BOADICEA (95% confidence interval [CI]: 0.68–0.72), 0.71 for IBIS (95% CI: 0.69–0.73), 0.68 for BRCAPRO (95% CI: 0.65–0.70) and 0.60 for BRCAT (0.58–0.62) [71]. Assessment of calibration was limited to the O/E ratio: BOADICEA 1.05 (95% CI: 0.97–1.14), IBIS 1.03 (95% CI: 0.96–1.12), BRCAPRO 0.68 (95% CI: 0.65–0.70), and BCRAT 0.79 (95% CI: 0.73–0.85).

Recently, there has been increasing interest in ‘machine learning’ prediction modelling for healthcare. Whilst arguably perceived to be more flexible (e.g. better at capturing non-linear, complex interactions), less reliant on assumptions than traditional regression and capable of handling some forms of data that regression models cannot, machine learning has not been shown to be inherently better than traditional statistical modelling approaches [72]. Datasets used for ML modelling should capture clinical reality, i.e. reflect the target population, and the architecture of any algorithm should be reported, given their structural flexibility. Clarity of reporting model development can be problematic [73] and validation/performance assessment approaches may not always be appropriate or transparent, especially when comparing different approaches. One recent study compared the performance of the BOADICEA model with a Markov chain Monte Carlo generalised linear mixed model, an adaptive boosting model and a random forest model developed using data from a single oncogenetic institution in Switzerland that focusses on counselling and testing for hereditary cancer syndromes [51]. Whilst the machine learning models were declared to outperform BOADICEA, no robust evaluation of model calibration was performed, and the effective comparison was an external validation of BOADICEA versus an internal validation of the new model using the data they were derived from. This used cross-validation with a low number of repeats (*n* = 20), which presumably was used for hyperparameter tuning as well as performance evaluation (not elaborated in paper), an option that is optimistically biased [74, 75]. Further work in this area of comparing different model-building strategies for predicting risk should focus on more robust, meaningful comparisons.

Overall, a range of clinical prediction models has been developed that could be used to guide risk-stratified screening, some of which are undergoing evaluation in trials of personalised screening. The ability of models to guide risk-stratified screening by predicting incident breast cancer risk in asymptomatic women is uncertain [64], even if integrating clinical, genetic and imaging-derived variables [30, 47, 49, 68]. There is no single accepted benchmark for a given performance metric to render a model suitable for guiding personalised screening, and decisions regarding optimal models should not be made on a single metric. Instead, models need to be robustly assessed in terms of discrimination, calibration and potential clinical utility in the target populations. Whilst a model with an AUC of 0.5 in its target population cannot be informative, a high AUC in a model development/evaluation study does not guarantee utility in guiding risk-based screening. Poorly calibrated models may cause harm, and those with unstable performance across sub-groups may raise concerns regarding ‘algorithmic fairness/bias’. Some of these models are not fully developed using individual-level data, rather, are pre-determined systems of weights that are then applied to a test dataset to assess performance [30, 47]. Crucially, however, weak or non-existent calibration assessment, non-examination of performance heterogeneity, or the lack of consideration of geographical and temporal transportability [55, 76] are notable limitations. The QCancer (Breast) [50], IBIS [47] and iCARE [77] models are some examples wherein exploration of performance heterogeneity is performed according to age groups or other clinically relevant sub-populations (see Table 2). It has also been suggested that in order to minimise harms from overdiagnosis of indolent tumours, modelling the risk of developing lethal breast cancers could be more appropriate than modelling the diagnosis of any breast cancer in order to risk-stratify screening [11]. This is another avenue for further exploration and analysis.

### Epidemiological analyses and retrospective evaluations of risk-stratified screening

The real-world effects of implementing risk-stratified screening strategies [78] warrant evaluation in prospective studies and trials. Whilst trials have recently been initiated (see later section), there have also been several explorations of the possible benefits and harms of using epidemiological approaches in a jurisdiction where multiple forms of mammography screening are available, namely in Taiwan. This complements studies modelling breast cancer risk in large cohort studies relative to age, or retrospectively simulating the effect of implementing different screening practices in screening cohorts. Table 3 summarises the evidence from such epidemiological papers or retrospective clinical evaluations simulating the possible effects of risk decision rules using patient data.

**Table 3:** Comparison of studies evaluating risk-stratified screening using simulations on retrospective data, or epidemiological studies.

Study	Country and setting	Description of modelling processes	Key results
Retrospective evaluations using clinical data, or epidemiological modelling			
van den Broek et al. [178]	US Women aged 30–50 years	Breast cancer simulation models: average-risk women, screened according to USPSTF guideline Family history strategy Polygenic breast cancer risk model (313 SNPs) Family history + polygenic risk	*Per 1000 women screened, during lifetime:* 118 life-years gained, 7 deaths averted, 15 overdiagnoses, 920 false positives 125 life-years gained, 6.9 deaths averted, 14.9 overdiagnoses, 1000 false positives 141 life-years gained, 7.4 breast cancer deaths averted, 16.0 overdiagnoses, 1156 false positives 154 life-years gained, 7.9 deaths averted, 16.6 overdiagnoses, 1169 false positives
Mukama et al. [82]	Sweden, *n* = 5,099,172	10-year cumulative risk curves for breast cancer Analysed risk levels of women with parity and first birth age: risk-adapted starting age of screening based on reproductive profiles	Women with first birth at age <25 years and one child attained the same level of risk as average 50-year-old female at age 51 years; those with parity of 4 or more met this threshold at 59
Mukama et al. [81]	Sweden, *n* = 5,099,172	Modelled age at which women with specific permutations of family history variables attained the risk level of the average 50-year-old woman (age at which screening starts)	If screening would be advised to start at 50 years, women with one first-degree relative diagnosed with breast cancer aged <40 years met the benchmark level of risk at 36 years of age
Lee et al. [88]	US Screening mammograms from 2,647,315 women	Separated women into risk groups based on 5-year age bracket, family history of breast cancer, personal history of breast cancer and dense breasts	Women aged 30–34 years had similar cancer detection rates and recall rates as those aged 40–49 years, suggesting earlier screening in women at higher risk may be appropriate
Burnside et al. [87]	US Screening mammograms from 10,280 women	Cross-sectional study comparing two scenarios: standard age-based screening versus risk-based, defined as having 5-year risk greater than average 50-year-old	Age-based screening diagnosed more cancers than risk-based (68 versus 26%), more false positives (50.3 versus 12.1%)
Prospective epidemiological studies			
Yen et al. [79]	Taiwan, population-based cohort study, *n* = 1,429,890	Cohort study of three screening strategies, adjusting for propensity score for participation: clinical breast examination, risk-based mammography and universal mammography (aged 50–69 years)	No overdiagnosis compared to clinical examination for risk-based screening, versus 13% overdiagnosis with universal screening 41% reduction in breast cancer mortality with universal screening (adjusted for year of birth and propensity score), non-significant reduction with risk-stratified screening

A large Taiwanese study (*n* > 1.4 million) exploited the natural experiment of the concurrent availability of three screening approaches in the country’s population, namely annual clinical breast examination as the baseline (women aged 35 years and over), risk-stratified biennial mammography screening or universal mammography (both for women aged 50–69 years) [79]. The existence of three available approaches was predicated by a low breast cancer incidence rate in 2002–2004 and concerns about healthcare system capacity for whole population screening at the time, although the rates have increased since [80]. Risk stratification used a ‘risk score’ derived from reproductive/menstrual history and family history data obtained during attendances for clinical breast examination between 1999 and 2001, with the median of the risk scores used as the cut-off for eligibility for biennial mammography. Using propensity score methodology to try to adjust for disparities in baseline risk factors across the three groups (age at menarche, parity, breastfeeding and BMI), Cox models were used, where screening modality was modelled as a time-dependent covariate. Compared to clinical examination, universal biennial mammography had a higher breast cancer detection rate, was associated with a downwards stage migration of detected cancers, a 13% overdiagnosis rate (95% CI: 8–18%), a 30% reduction in stage II + breast cancers (hazard ratio [HR] 0.70, 95% CI: 0.66–0.74), and a 41% reduction in breast cancer mortality (95% CI: 27–52%) when adjusting for propensity score and year of birth [79]. Compared to clinical examination, the overdiagnosis with risk-based screening was negligible (HR for diagnosis 0.97, 95% CI: 0.92–1.03), there was an 8% reduction in stage II + breast cancers (HR 0.92, 95% CI: 0.86–0.99) and a ‘non-significant’ reduction in breast cancer mortality of 14% (HR 0.86, 95% CI: 0.73–1.03) [79]. However, the risk-stratification mechanism in this study was unclear—data were not provided on the modelling methodology used, the risk score covariates, the risk score distribution in the population seeking to opt into risk-based screening (or across the three arms) or performance evaluation to assess if this approach was suitable for clinical use. Further detail is needed to make meaningful inferences on the performance of risk-based screening versus ‘standard’ screening in this analysis. In addition, given the relatively low, albeit increasing, breast cancer incidence rate in this population, risk stratification beyond age and sex may have different proportional benefits in Taiwan in comparison to other nations.

Rather than analysing the effects of altering screening intensity or avoiding screening in low-risk women, a large Swedish cohort with linkage to several national databases (*n* > 5,000,000 women) was used to assess whether earlier screening starting ages could be appropriate for some women. By using 10-year cumulative risk estimations, the risk level of the ‘average’ 50-year-old woman that would be offered screening was calculated as a benchmark. The ages at which other women would attain the same 10-year risk were compared, based on patterns of family history [81] or personal reproductive history (parity and age at first birth) [82]. Both studies found that either approach could identify women who, despite not being eligible to start age-based screening, had the same 10-year risk estimate as 50-year-old women who would be invited to screen, or indeed may only attain that same threshold of risk after age 50 years. For example, women who had their first baby aged under 25 years met the benchmark aged 51 years, whereas women who had four births by age 25 years met this at 59 years of age [82]. Furthermore, women with one first-degree relative diagnosed with breast cancer before the age of 40 years met the average risk of women starting age-based screening at age 36 years [81]. Therefore, despite debate around the benefits of universally expanding screening to younger age groups such as the lack of long-term effect seen in Age UK [83], selected women with selected risk factors may be suitable for earlier or delayed commencement of early detection strategies. The optimal way to assess risk would require elucidation as reliance on two albeit important risk factors may inadequately capture risk.

In the radiological literature, some commentators have voiced criticism of the potential harms offered by risk-stratified screening [84–86], typically fuelled by retrospective studies applying risk factor-based decision rules to cohorts of women that partook in service screening [86–89]. For example, Lee et al. examined recall rates, cancer detection rates and positive predictive values for biopsy recommendation and the fact of biopsy across age groups, when accounting for breast cancer family history, personal breast cancer history and having dense breasts in a cohort of >2.6 million women [88]. The recall and cancer detection rates in 30–39-year-old women were the same with these risk factors undergoing incidence screening as the 40–49-year-old ‘average-risk’ women undergoing screening; thus, they concluded that such a higher-risk women may benefit from earlier screening starting age. Other institutional studies have expressed concern that risk-stratified approaches have the potential to miss 75.6–88% of cancers if screening was purely based on family history, 56–86% of cancers if density was the sole determinant or 43.5–76% if access to screening was dictated by positive family history and breast density [86, 89]. Such approaches are not powered to assess the effects of varying screening approaches on stage at detection nor breast cancer mortality, but most crucially, they rely on relatively simplistic determinations of relative risk. Prospective clinical trials are not evaluating such approaches, rather, more nuanced methods for estimating risk.

Overall, currently available epidemiological evaluations or retrospective clinical estimations of breast cancer screening guided by individualised risk are insufficient to inform the utility of risk-stratified screening.

### Prospective cohort studies

Three notable cohort studies are currently exploring multiple aspects pertaining to the feasibility and acceptability of personalised risk assessment in the general population: specifically, the Personalised RISk-based MAmma screening study (PRISMA), the Karolinska Mammography Project for Risk Prediction of Breast Cancer (KARMA) and the Predicting the Risk of Cancer at Screening study (PROCAS).

PRISMA is a Dutch collaboration between institutions including Radboud UMC Nijmegen and the North, East, West and South Screening Programmes. In 2014, PRISMA started recruiting asymptomatic women aged 50–75 years in the general population eligible for the national screening programme for data collection via questionnaires, blood and saliva samples and mammograms for assessing breast density. It has a target of 90,000 women with regards to risk factor questionnaire data collection and imaging, and 27,000 blood samples. It aims to not only develop risk prediction models as a fulcrum for investigating risk-based screening strategies but undertake a robust assessment of the acceptability of risk-based screening from ethical, psychological, legal and logistical perspectives. Outputs from PRISMA thus far include multi-cohort qualitative research incorporating individuals from KARMA and PROCAS, which identified a preference for risk-tailored assessment results communication (e.g. letters for below average and average risk, face-to-face appointments for higher risk), the need for standardised risk assessments within national policies and detailed information needs for women in different European countries [90, 91].

The KARMA prospective screening cohort is developing an extensive resource of banked biological, mammogram and lifestyle/clinical factor information from over 70,000 women [92]. Its aims include identification of novel circulating risk markers, genetic risk factors and imaging protocols, assessment of high-throughput breast density measurement, trials of pharmacological prevention therapies such as lower-dose anti-oestrogen therapy and risk communication as well as the development of new risk prediction models with an evaluation of how these could be implemented within screening routines. Notable outputs include the CAD2Y model, which integrates mammographic features such as computer-detected microcalcifications with ‘clinical’ factors for short-term risk prediction [93], risk estimation integrating mammographic density and polygenic risk [48, 94], and contributions to the identification and understanding of genomic breast cancer risk by multi-centre consortia [95–98].

The PROCAS 2 study began in 2015 following PROCAS 1, which recruited over 50,000 women eligible for screening mammography at the Great Manchester NHS Breast Screening Programme. Lifestyle, reproductive history and other clinical informations were collected via questionnaires, with imaging assessment of mammographic density and DNA obtained for polygenic risk analysis [99]. Numerous studies have been undertaken within the remit of PROCAS, such as the evaluation of Tyrer–Cuzick and Gail risk prediction models in a screening population, the predictive impact of the inclusion of mammographic density [68] and/or polygenic risk score components into these [30, 100], extensive assessment of risk feedback and perception [101–103] and probing associations between ethnicity and mammographic density [99]. PROCAS outputs have been central to supporting the feasibility of population-based breast cancer risk assessment [101, 104], including identifying no major psychological harms of providing 10-year risk estimates from different forms of risk algorithms [103].

### Prospective evaluations and trials of risk-stratified screening

Prospective clinical studies and ideally randomised trials should evaluate risk-based screening practices in terms of outcomes, balance of harms and benefits, cost-effectiveness and acceptability. Several key studies are underway [105–108], including NCT04359420 [108]. This is a non-randomised, counterbalanced study across seven screening sites in the United Kingdom, in which women on an invitation to the NHS Breast Screening Programme will either be offered the standard programme or the additional invitation to use BC-Predict, an automated system for offering a breast cancer risk assessment (to include questionnaires, breast density measurement and polygenic risk) on an invitation to screen [108]. Its aims include assessing risk assessment uptake after offer, uptake of risk consultation, chemoprevention or additional mammography, as well as risks of potential cancer worry, anxiety or health service costs [108]. Many others do not strictly seek to evaluate the outcomes of screening intensity/eligibility decisions based on individualised risk estimation. Indeed, some are also exploring how best to communicate personal risk (e.g. PROSPR/PCIPS 3, NCT01879189), promote breast screening uptake based on risk factor-specific educational materials (e.g. NCT00416975) or identify optimal imaging modalities for women at specific levels of predicted risk (e.g. NCT00003736).

The Women Informed to Screen Depending on Measures of risk (WISDOM) trial is a preference-tolerant randomised trial of a risk-based screening algorithm versus standard screening practice in the United States that started in 2016 [105]. Absolute breast cancer risk estimates are generated using the BCSC model [49], modified by a polygenic risk score incorporating 96 SNPs using Bayesian principles [105] and testing for nine high- or moderate-risk genes (*BRCA1*, *BRCA2*, *TP53*, *STK11*, *PTEN*, *CHD1*, *ATM*, *PALB2* and *CHEK2*). Predicted risks at 5 years and age dictate the screening strategy. For those aged 40–49 years: women with a 5-year risk of <1.3% are not being offered screening, those with a 5-year risk of 1.3% or greater are undergoing biennial mammography, whilst women with extremely dense breasts or who are carriers of *ATM/PALB2/CHEK2* mutations without a positive family history are undergoing annual mammography. For women aged 50–74 years, all are undergoing biennial mammography unless they are carriers of *ATM/PALB2/CHEK2* mutations without a positive family history in which case they receive annual mammography. Regardless of age group, annual mammography with adjunct MRI is being deployed in carriers of *BRCA1/BRCA2/TP53/PTEN/STK11/CDH1* mutations regardless of family history, carriers of *ATM/PALB2/CHEK2* mutations with a positive family history, those who had chest irradiation between the ages of 10–30 years or have a 5-year breast cancer risk of at least 6%. With target recruitment of 100,000 women, it has been projected that 75% of women aged 40–49 years will be allocated to ‘no screening’, whereas 91% of women aged 50–74 years will undergo biennial mammography [105]. The primary endpoints are non-inferiority to standard screening regarding the number of late-stage breast cancers diagnosed (>stage IIB), and rates of recall and breast biopsy. Secondary endpoints include the rate of stage IIB and interval cancers, recall rates, rates of DCIS diagnosis, rates of chemoprevention use, cancer incidence rate, PROMIS anxiety score and rates of systemic therapy use between arms. Importantly, the design is inherently adaptive so that risk assessment methodology and screening strategies are adjustable in line with future evidence under a ‘continuous improvement’ framework [18].

The population-based Tailored Breast Screening Trial (NCT02619123) was initiated in Italy in 2013, and is randomising pre-menopausal women aged 44 years and older to invitation to ‘tailored screening’ or an active comparator [107]. The target recruitment is 33,200 women and the estimated study completion date will be early 2022. In the tailored arm, those with BI-RADS grade C–D breast density receive annual mammogram invitations until age 50 years and then standard population screening; those with lower density breasts are invited 2-yearly until age 50 years and then standard population screening. In the active comparator arm, women are invited to annual mammography until age 50 years, followed by usual population screening. The primary outcome measures are the difference in cumulative interval cancers between arms (also by density group) and the cumulative incidence of >T2 or node-positive breast cancers by arms (also by density group). Secondary endpoints include a comparison of false-positive rates between arms, the cumulative incidence of all breast cancer cases and attendance to screening. However, the ramifications of this trial on clinical practice may be limited, due to the basis for stratification (dense versus non-dense breasts), the small divergences in screening strategy (annual or biennial mammography) and the short period in which the screening intensity will be altered.

The other key trial is My Personalized Breast Screening (NCT03672331); an international study seeking to recruit 85,000 women aged 40–70 years, in which screening strategy in the experimental arm will be dictated by risk assessment incorporating age, family history, previous benign breast disease, hormone/reproductive history and a polygenic risk score. Specifically, women with one or no first-degree relatives with breast or ovarian cancer will utilise the MammoRisk® model, otherwise, the Tyrer–Cuzick model will be used (see above). No data regarding the proprietary MammoRisk® algorithm structure itself or results of performance evaluation is accessible on the owning company’s website (https://www.predilife.com/en/home-2/, accessed 1 November 2020) or identifiable on Medline, although studies of acceptability/ease of software use are available [109, 110]. In the comparator arm, women will be screened with mammography, tomosynthesis or MRI in accordance with extant national guidelines, whereas in the active arm, an estimated 5-year risk will inform mammography and/or tomosynthesis screening every 1 to 4 years (with or without ultrasound depending on breast density). The trial has a non-inferiority design and the primary outcome is the incidence of stage >II breast cancers for the risk-stratified arm. Secondary outcome measures include a superiority analysis regarding the incidence rate of stage >II cancers, rates of false positives and benign biopsies, subject anxiety, health-related quality of life according to the EQ-5D and cumulative breast cancer diagnosis rates.

Overall, three key randomised trials are underway to assess the outcomes associated with different screening intervals, starting age, or imaging modalities based on individualised risk assessment, although there is diversity in the robustness of the risk assessment mechanism. The initial results from these trials are likely to emerge within the next 3 years, and it is notable that the largest and most comprehensive is adaptable [105], in that newer methods of risk assessment or amended screening strategies can be incorporated should novel evidence emerge.

### Health economic evaluations of stratified screening

Economic simulations of risk-stratified breast screening have modelled the clinical outcomes and cost-effectiveness of a range of scenarios in health systems such as the United Kingdom’s NHS Breast Screening Programme [16, 111], the United States [112, 113], Germany [114, 115], the Netherlands [116] and China [117]. Models have evaluated the stratification of screening intensity based on classical risk factors such as family history, class of breast density, age or relative risk based on polygenic risk scores. Across models, analytical approaches have been compared, e.g. relative numbers of breast cancer deaths avoided, rates of overdiagnosis, incremental costs or incremental quality-adjusted life-years and incremental cost-effectiveness ratios.

Whilst it appears that such modelling does lend support to the general concept of altering screening practices on the basis of risk to strike a more favourable balance of benefits (reduction in breast cancer deaths) and harms (overdiagnosis and unnecessary treatment) or to formulate a more cost-effective approach, a cohesive narrative regarding a particular algorithm-informed strategy is difficult to synthesise. Table 4 summarises the approaches and key results from such studies. These existing studies may diverge from real-world practice in terms of the ascertainment of individual risk (in terms of nuance and approach) or may be limited by incomplete information on risk distributions in the target population.

**Table 4:** Summary of health economic and outcomes models evaluating risk-stratified breast screening identified during the scoping review.

Study reference	Population modelled	Modelling approach utilised	Risk groups and screening scenarios simulated	Key results
Sankatsing et al. [116]	Netherlands—women without BRCA1/2 mutation Women born in 1974 Time horizon age 40–death	Microsimulation (MISCAN—microsimulation screening analysis; semi-Markov processes)	Risk groups: low, average and high, using ‘common risk factors’, excluding breast density Simulated: biennial screening aged 50–74 overall; biennial or triennial screening for low-risk women starting 50–60 to 64–74 years; annual or biennial screening for high-risk women starting 40–50 until 74–84 years Assumption of 100% screening attendance	Per 1000 women: *Biennial screening to all, 50–74:* 206 life-years gained, 16 deaths avoided, 187 false positives, 5 overdiagnosed cases *Triennial screening 50–71 for low risk:* 134 life-years gained, 10 breast cancer deaths avoided, 102 false positives, 3 overdiagnosed cases *Biennial screening 40–74 for high risk:* 380 life-years gained, 26 breast cancer deaths avoided, 371 false positives, 7 overdiagnosed cases
Arnold et al. [114]	Germany Women aged 50, followed to age 100 or death	Microsimulation Markov model	Risk factors: family history, personal history of biopsy, breast density Compared annual, biennial and triennial universal screening to risk-adapted strategies based on relative risk (three risk categories) Assumption of 54% adherence	Risk-stratified programmes may be more efficient, depending on mortality reduction or QALYs are strategic focus At 54% adherence, compared with no screening, screening women with relative risk >1 was projected to generate 8.63% mortality reduction, incremental QALYs of 0.023 and incremental costs of 211 Euros per woman (2017 prices).
Sun et al. [117]	China (urban population)	Prior natural history Markov model	High-risk defined: relative risk >2 High-risk women: screened using USS aged 40–44 years with subsequent mammography if indicated; with both modalities if 45–69 years Low-risk women: no screening (diagnosis after symptoms arise) Simulated complete treatment and also 70% treatment after diagnosis	*Compared with no screening, risk-adapted approach screening every 3 years, with full treatment:* Lifetime costs US$184 per case (2014 prices), 22.99 QALYs, 0.0127 difference in QALY *Compared with no screening, annual screening and full treatment:* Lifetime costs US$335.43 per case (2014 prices), 23.01 QALYs, 0.028 different in QALYs
Pashayan et al. [16]	UK Cohort *n* = 364,500 Women aged 50 years followed up to 85 years	Life-table model	Three cohorts with screening based on risk group: (1) No screening (2) Screen all women aged 50–69 years as per NHS BSP (3) Only women above risk polygenic risk threshold screened every 3 years from age 50–69 years	*Compared to no screening, risk-based screening:* Overdiagnosis:deaths prevented ratio increased from 0.07 to 0.99 as risk threshold lowered (from 99th to 71st percentile) Minimum ICER at 77th percentile of risk threshold (£11,911 per QALY gained), versus £66,445 when using 99^th^ percentile as risk cut-off (price date not specified) At 32nd percentile of risk, risk-adapted screening generated an incremental cost of £20,066 (price date not specified), 450 more QALYs, and 7 fewer breast cancer deaths
Gray et al. [111]	UK Women eligible for NHS BSP (aged 50–70 years)	Discrete event simulation	Four stratification methods in NHS BSP: (1) Absolute 10-year risk as per of Brentnall (BCR 2015): <3.5% triennial screening, 3.5–8% biennial screening, >8% annual screening (2) Relative 10-year risk: low tertile = triennial, middle tertile = biennial, high tertile = annual (3) Supplemental US for women with high breast density (4) Approach 1 plus the supplemental US as in (3)	*Compared to the current NHS BSP strategy (screening 50–70 years of age ever 3 years):* Risk-stratification methods 1 and 2 were deemed cost-effective relative to threshold range of £20,000–30,000 per QALY ICER for method 1 versus UK BSP: £16,689 (2015 prices) ICER for method 2 versus UK BSP: £23,924 (2015 prices)
Trentham-Dietz et al. [112]	US Women aged 50+ Lifetime horizon	Microsimulation models ×3	Examined various combinations of breast density (four categories) and relative risk for factors other than density (1.0, 1.3, 2.0 and 4.0) Settings of annual/biennial/triennial screening for women aged 50–74 years, and also for women 65–74 years Assumed 100% adherence	*Per 1000 women with fatty breasts/scattered fibroglandular density* + *RR 1 or 1.3:* Biennial screening (50–74): 5.1 deaths averted Triennial screening (50–74): 3.4 death averted Biennial screening (50–74): 4.1 deaths averted Triennial screening (65–74): 6.5 deaths averted Triennial screening for average-risk women with low-density breasts provided favourable balance of harms and benefits and is cost-effective Annual screening for higher risk (RR 2.0 or 4.0) with heterogeneously or very dense breasts has favourable balance of benefits and harms and is cost-effective
Schousboe et al. [113]	US Women aged 40–49, 50–59, 60–69 and 70–79 (initial mammography at 40) Lifetime horizon	Markov cost–utility microsimulation model	Modelled risk based on BI-RADS breast density category, and up to 2 risk factors (family history or previous biopsy) Examined annual, biennial, triennial, 3–4 yearly mammography or no mammography	A range of cost-effective strategies for women of different age groups, breast density and presence of up to 2 risk factors were identified (assuming $100,000 and $50,000 cost-effectiveness thresholds), e.g. at a 50,000 cost-effectiveness threshold: BI-RADS B-D, or BI-RADS A + 1–2 risk factors: biennial screening 50–59 years, reassess at age 60 BI-RADS A + 0/1 risk factors: 3–4 yearly mammography 50–59 years, reassess at age 60

*BI-RADS* Breast Imaging-Reporting and Data system classification, *USS* ultrasound scan, *NHS BSP* National Health Service Breast Screening Program (in the United Kingdom), *ICER* incremental cost-effectiveness ratio, *QALY* quality-adjusted life year.

More simplistic estimations of relative risk on a small number of covariates tend to be used as the stratification mechanism, such as ‘positive family history’ or breast density category, or use blunter approaches to ascertain ‘high-risk’ women, such as the relative risk of 2.0 or greater. Multivariable risk assessment seeks to offer more nuanced estimations of risk, which are not recapitulated in many studies. Modelling the implementation of a polygenic risk assessment to inform relative risk may be limited by the relatively small absolute contribution that genetic factors may play in the majority of women that do not have high- or medium-penetrant mutations. As some studies have highlighted, the lack of data on the true distribution of risk groups in the target population as a whole limits their assessment of the impact of risk-based screening practices in a health system.

The approaches to and thresholds used to risk-stratify screening appear to differ between studies. Some seek to find the economically optimal screening interval or starting age for screening in cohorts simulated as having set risk levels, or even have intensified screening with additional imaging modalities as one pathway in their models. Few compare existing age-based methods with risk-stratified approaches in the same target population, and fewer evaluate a more robust view of risk-adapted screening, namely not offering screening to those at the lowest risk. Furthermore, the direct comparator in some studies is ‘no screening’ rather than an evaluation of transitioning from age-based screening to truly risk-adapted screening.

### Qualitative research

In order to accept screening strategies tailored to individual risk, women need to be able to access and comprehend accurate risk estimations [118–120], and indeed, many women are interested to understand and discuss their risk [90, 101, 102, 121, 122]. Risk communication and risk perception are multifaceted and complex [119], yet it is striking that as few as 10% of women have accurate perceptions of personal risk with an otherwise roughly even split between under- and over-estimators [123, 124]. The variable use of absolute and relative risks can have major influences on screening intentions or in some cases be misleading [125].

A growing body of qualitative research has synthesised evidence from focus groups, semi-structured interviews and other methodologies regarding stakeholder views on the implementation and acceptability of risk-stratified screening [90, 126–136]. Particularly key considerations are those pertaining to communication of risk and risk-based pathways across languages and cultural groups [127], socioeconomic groups and those that have lower engagement with preventive healthcare. Generally, perceptions of risk-based screening appear to be favourable, whether based on genetic risk [133, 137] or other factors. Whilst it felt to be acceptable in principle by many women, the evidence base used to support these approaches needs clear articulation to secure buy-in [126] and reasons for heterogeneous policies for different groups need to be transparent [126]. Importantly, perceptions of risk stratification as a euphemism for service funding reductions may arise [128], and there should be cognisance of anxiety around self-directed risk assessment if used, such as via websites or apps [138] (albeit, at low levels) [134].

## Discussion

Genetic, pharmacological and environmental or lifestyle factors affect breast cancer risk, with heterogeneity encountered in terms of individual women’s risks and the risks posed by individual tumours. As one-size-fits-all approaches are increasingly disparaged in breast cancer treatment, detection and prevention under the auspices of ‘personalised healthcare’ [21], the use of age as the single precision factor for guiding early detection strategies in women may be over-reductionist [19–21]. Approximately one-quarter of all breast cancers are diagnosed in women ineligible for screening due to age, only around 30% of breast cancers in the United Kingdom are detected by the triennial UK screening programme [139] (43% of all breast cancers in the United States [140]), and current methods may predicate overdiagnosis [3, 5, 8, 11]. In the United States, 73% of breast cancers in biennial screeners are detected by mammography, and 78% of breast cancers are detected by screening in annual screeners [141]. Approaches that tailor screening intensity and personalise modalities to those that stand most to gain and minimise unnecessary interventions in those with little to gain require robust evaluation.

It is relevant to distinguish between the use of genetic, lifestyle and other factors in tools for long-term risk prediction to guide imaging strategies over a woman’s ‘screening lifetime’ and estimating the risk of an underlying cancer being present at the time of screening. Compared to settings such as lung cancer screening, where smoking history has the predominant effect on decision-making and may approach immediate diagnostic relevance, the long-term prediction of breast cancer relies on appropriate multi-factorial assessment using data points that have ‘weaker’ effects on risk.

Approaches to risk-stratified screening that have been explored include altering screening intensity, screening starting age or screening technology used and therefore a single consensus definition has not emerged, nor on the optimal form risk-stratified screening could take. One concern is that a paradigm withholding access to screening with a sensitive imaging modality in those deemed ‘low risk’ by a less sensitive predictive model may be inappropriate. As such, there must be judicious consideration of whether a ‘screen-only high-risk’ model is ethically, economically and clinically appropriate in comparison to other models such as the current effective approach, or those that seek to deliver screening that is ‘tailored according to risk’, where risk assessment influences the factors mentioned above without removing women from the screening pool. It is not yet clear which if any of the currently available risk models or risk-based strategies deliver on this concept—ongoing trials and other continued progress in optimising individualised risk estimation and screening strategy should begin to deliver clinically informative answers in the coming years [105–107]. Further, individualised risk estimation may not only be useful for stratifying screening strategy to reduce harms but to also reduce the substantial public health burden of breast cancer, potentially through identifying women at previously unknown high risk suitable for prevention therapy (e.g. Anastrozole or Tamoxifen).

Alongside the elucidation of the optimal degree of nuance for breast screening pathways, it would be essential to explore risk communication strategies, and continually monitor such programs’ effectiveness both clinically and economically. Another avenue for future work will be further assessment of stated preferences, such as through discrete choice experiments [142, 143] or contingent valuation studies, and the trade-offs between benefits, risks and costs in making such preferences in a stratified programme.

Concerns have been raised that risk-stratified screening poses the danger that many breast cancers may be ‘missed’ by not screening women believed to be at low risk [84, 144]. However, many such studies ignore the inherently multivariable nature of the best performing currently available risk models [30, 47, 50], compare current age-based screening against a straw man of blunt risk estimation that is not advocated, and are not powered to identify the effects on important outcomes such as the proportion of late-stage cancer detected, or cancer survival. Stratification may lead to changes in imaging strategy, such as increased use of supplemental MRI, which randomised trial evidence suggests reduces rates of interval cancers in women with extremely dense breast tissue [145]. Recent evidence of supplemental abbreviated MRI in women at average risk with dense breasts and negative digital tomosynthesis results appears to increase the prevalent cancer detection rate (up to 27.4 per 1000 women), but the survival benefits are yet to be quantified [146].

Overdiagnosis and overtreatment have been widely acknowledged in prostate cancer screening for years [147–150], yet a progression towards risk-stratification of screening itself or risk-guided management of detected neoplasms is far more mature than in the breast cancer field [149, 151]. The debate around differing estimates of screening mammography’s benefits and harms has become increasingly polarised over recent decades [4, 18, 152–158], with consistent disagreement over the interpretation of decades-old trials, the reliability of specific randomised studies, statistical approaches used to interpret them or epidemiological studies [11, 158–160]. However, whilst the clearly prevailing consensus is that screening mammography reduces breast cancer mortality, whether this can be further improved is a worthy avenue to explore. Recently, trials have been designed to provide evidence to inform this. Should their results be positive, they must be followed by careful consideration of whether the ‘new normal’ would be acceptable to healthcare systems, policymakers, clinicians and members of the public.

## Data Availability

No original data were generated for this review. The literature search strategy is provided.
